# Development and Validation of the Individualized Prognostic Nomograms in Patients With Right- and Left-Sided Colon Cancer

**DOI:** 10.3389/fonc.2021.709835

**Published:** 2021-11-01

**Authors:** Zai Luo, Zhongmao Fu, Tengfei Li, Yuan Zhang, Jianming Zhang, Yan Yang, Zhengfeng Yang, Qi Li, Zhengjun Qiu, Chen Huang

**Affiliations:** ^1^ Department of Gastrointestinal Surgery, Shanghai General Hospital, Shanghai Jiaotong University School of Medicine, Shanghai, China; ^2^ Institute of Translational Medicine, Shanghai General Hospital, Shanghai Jiaotong University School of Medicine, Shanghai, China; ^3^ Department of Medical Oncology, Shuguang Hospital, Shanghai University of Traditional Chinese Medicine, Shanghai, China

**Keywords:** tumor-stroma percentage, nomogram, colon cancer, location, prognosis

## Abstract

**Background:**

The overall survival (OS) of patients diagnosed with colon cancer (CC) varied greatly, so did the patients with the same tumor stage. We aimed to design a nomogram that is capable of predicting OS in resected left-sided colon cancers (LSCC) and right-sided colon cancers (RSCC), and thus to stratify patients into different risk groups, respectively.

**Methods:**

Records from a retrospective cohort of 577 patients with complete information were used to construct the nomogram. Univariate and multivariate analyses screened risk factors associated with overall survival. The performance of the nomogram was evaluated with concordance index (c-index), calibration plots, and decision curve analyses for discrimination, accuracy, calibration ability, and clinical net benefits, respectively, which was further compared with the American Joint Committee on Cancer (AJCC) 8th tumor-node-metastasis (TNM) classification. Risk stratification based on nomogram scores was performed with recursive partitioning analysis.

**Results:**

The LSCC nomogram incorporated carbohydrate antigen 12-5 (CA12-5), age and log odds of positive lymph nodes (LODDS), and RSCC nomogram enrolled tumor stroma percentage (TSP), age and LODDS. Compared with the TNM classification, the LSCC and RSCC nomograms both had a statistically higher C-index (0.837, 95% CI: 0.827–0.846 and 0.780, 95% CI 0.773–0.787, respectively) and more clinical net benefits, respectively. Calibration plots revealed no deviations from reference lines. All results were reproducible in the validation cohort.

**Conclusions:**

An original predictive nomogram was constructed and validated for OS in patients with CC after surgery, which had facilitated physicians to appraise the individual survival of postoperative patients accurately and to identify high-risk patients who were in need of more aggressive treatment and follow-up strategies.

## Background

Colon cancer (CC) is among the most common malignancies in the gastrointestinal tract, with an estimated annual incidence of 1.09 million cases and 551,268 death cases worldwide (1). Despite more tumor biology characteristics and potential prognostic factors were found, prognosis prediction of primary CC mainly depended on tumor-node-metastasis (TNM) status in the diagnosis (2, 3). TNM staging system is a common criterion, as recommended by the American Joint Committee on Cancer (AJCC), to predict the outcomes of CC patients by evaluating tumor size (T), regional lymph-node involvement (N), as well as the presence of distant metastases (M) (4). Due to heterogeneity of CC and its incompetence in assessing the metastatic potential of CC, TNM system is not capable of predicting outcomes of all CC, thus which cause survival paradox (5, 6). For example, patients with positive lymph nodes (N+) were classified into stage III, regardless of T stage, while patients with early T stage and N+ obtained better outcomes than patients with high T stages and negative lymph nodes (N−) (6). Namely, relying solely on the TNM stage was not enough to predict prognosis and determine treatment strategy of CC patients, which might have caused under- or overtreatment (7). Thus, there is ever-increasing need to identify novel robust prediction tools alongside current TNM stages.

Exactly, to remedy the deficiency of the TNM classification system, accumulating prognostic markers including other clinical, pathological parameters and diverse genes have been explored, verified, and applied in clinical practice (8). Recent evidence suggested tumor stroma percentage (TSP) and log odds of positive lymph nodes (LODDS) were practicable determinants in several solid tumors including colorectal cancer and gastric cancer (9, 10). TSP was a straightforward measure that can be assessed by microscopic inspection of hematoxylin and eosin (H&E)-stained tissue sections (9). TSP was defined as the proportion of stroma in the entire tumor tissue, and yielded prognostic information in colorectal cancer in recent studies (9, 11). LODDS was recently validated as an independent prognostic factor in colorectal cancer (CRC), which played a decisive role in prognostic assessment regardless of lymph node status and count (10).

Right-sided colon cancers (RSCC) were commonly found in the cecum, ascending colon, hepatic flexure, and/or transverse colon, while left-sided cancers (LSCC) were in the splenic flexure, descending colon, and/or sigmoid colon (12). Studies verified that RSCC and LSCC were differed in embryonic origin, anatomy, physiology, pathological type, and molecular biology. It thus concluded that RSCC and LSCC were recognized as two distinct entities in general (13–16). Nomogram, a simple statistical prediction tool, which contains multiple variables and achieves a high prediction accuracy in a specific event, has shown a more effective prognosis ability than traditional TNM staging systems in multiple types of cancers (17). However, previous nomograms were based on analysis of cohorts which mixed RSCC and LSCC together (18), lacking specific nomograms that respectively predicted the prognosis of RSCC and LSCC.

In this study, we investigated the clinical significance of TSP and LODDS in RSCC and LSCC, respectively, and validated their prognostic value. In addition, we systemically and comprehensively constructed two novel nomograms for RSCC and LSCC to avail clinicians of a more precisely survival rate and customizable treatment decisions.

## Materials and Methods

### Patients and Data Collection

This retrospective analysis was conducted in accordance with the Declaration of Helsinki. The study’s protocol was approved by the Clinical Research Ethics Committee of Shanghai General Hospital. Due to the retrospective nature of this study and anonymous use of patients’ data, informed consent was not required.

**Figure 1:**
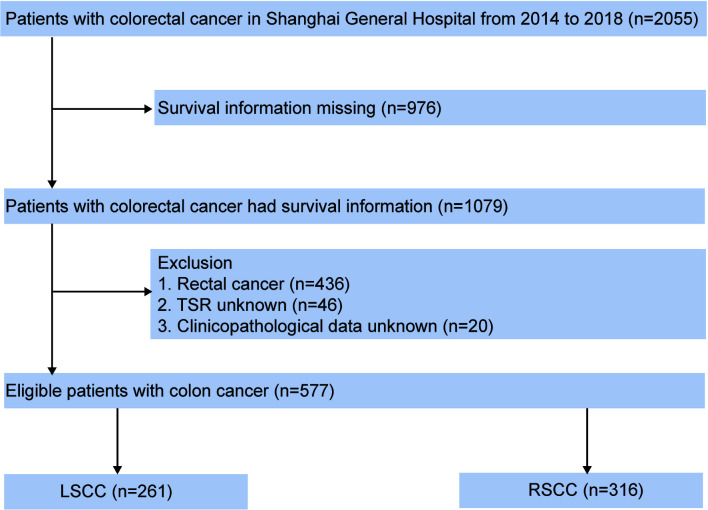
Flowchart of CC patients in our study.

In the present study, a total of 1,079 colon cancer cases diagnosed pathologically were enrolled from Shanghai General Hospital between January 2014 and December 2018. All patients had received laparoscopic colectomy. The flow chart of case inclusion and exclusion is shown in **Figure 1**. The detailed inclusion criteria were shown as follows: (1) patients who underwent laparoscopic colectomy as initial treatment and did not receive any preoperative treatment; (2) patients had pathology-confirmed CC diagnosis; (3) patients with complete clinicopathological and follow-up data. In addition, patients were excluded if they met the following exclusion criteria: (1) Absence of important clinicopathological factors, such as TSP and LODDS. (2) Incomplete survival information (survival months and survival status). TNM stage was evaluated according to the 8th edition of AJCC TNM classification. Demographic and clinicopathological data, including age, gender, TNM stage, positive lymph node, LODDS, tumor location, tumor size, Ki-67, carbohydrate antigen 72-4 (CA72-4), carcinoembryonic antigen (CEA), carbohydrate antigen 19-9 (CA19-9), carbohydrate antigen 50 (CA50), carbohydrate antigen 12-5 (CA12-5), TSP, caudal type homeobox 2 (CDX2), mutL homolog 1 (MLH1), mutS homolog 2 (MSH2), ERCC excision repair 1 (ERCC1), non-metastatic protein 23 (NM23), cytochrome c oxidase subunit II (COX2), hepatocyte growth factor receptor (c-MET), survival months, and survival status were retrieved. The details about primary antibodies in immunohistochemical (IHC) stains are as follows: Ki-67 (clone GT209429/11; Gene Tech, China; dilution 1:200, retrieval solution—pH 8), CDX2 (clone GT201929; Gene Tech, China; dilution 1:100, retrieval solution—pH 8), MLH1 (clone GT230429/11; Gene Tech, China; dilution 1:200, retrieval solution—pH 8), MSH2 (clone GT231021/29/11; Gene Tech, China; dilution 1:100, retrieval solution—pH 8), ERCC1 (clone GT215529; Gene Tech, China; dilution 1:200, retrieval solution—pH 8), NM23 (clone GT202621/29; Gene Tech, China; dilution 1:400, retrieval solution—pH 6), COX2 (clone GT211329; Gene Tech, China; dilution 1:200, retrieval solution—pH 8), and c-MET (clone Ab51067; Abcam, UK; dilution 1:250, retrieval solution—pH 6). All CC patients were advised to receive regular follow-ups after radical surgery according to clinical guidelines. Patients were generally followed up every 3 months in the first 2 years and every 6 months thereafter 3 to 5 years. Overall survival (OS) was defined as the time interval between the date of operation and the date of the death from any cause. For patients alive, the last follow-up date was July 2019.

### Construction and Validation of the Nomogram

Enrolled colon cancer patients from our database were identified and randomly divided into the training set of 462 patients and the internal validation set of 115 patients through a random number list generated by the R function “createFolds” to ensure that outcome events were distributed randomly between the two cohorts. The classification of categorical variables was determined by their clinical significance, which had been divided before the construction of the nomogram. In the training set, twenty characteristics were investigated by Kaplan-Meier curves and log-rank tests, and independent prognostic factors related to OS were identified by univariate and multivariate Cox regression analyses. Meanwhile, the impact of independent prognostic factors on OS were measured by hazard ratio (HR). Based on the significant factors, predictive nomograms for predicting OS were constructed by R software version 2.13.2 (http://www.r-project.org/).

Nomogram validation included discrimination and calibration curves. First, discrimination performance of the proposed nomogram was evaluated by concordance index (C-index), which value greater than 0.750 was considered to represent the relatively great concordance between the predicted and the observed responses (19). Second, calibration curves were performed by comparing the nomogram predicted OS probability with corresponding actual survival OS probability through the Kaplan-Meier method. In addition, decision curve analysis (DCA) and the receiver operating characteristic (ROC) curve were both applied in this study. DCA was used as a novel method to assess the nomogram’s ability in visualizing the clinical outcomes and evaluating the risk of adverse outcomes of individuals (20). ROC curve was used to compare the discriminative power of the proposed nomogram with the 8^th^ AJCC TNM classification. All analyses were performed using SPSS version 20 (IBM, Armonk, NY, USA) and R version 2.13.2 *via* the design and survival packages. A *P* value of <0.05 was considered as significant.

### Assessment of the TSP

The deepest point of tumor invasion of the H&E-stained sections of surgical biopsies were used to assess TSP. First, scanning of tumor sections was carried out using the automatic digital slice scanning system (KF-PRO series) at objective magnification ×10, and visualization was performed by the digital slice reading software K-Viewer (Konfoong Biotech, NB, China, 1.5.3.1). Given that there was some heterogeneity in assessment of TSP among biopsy sections, a representative region with the most invasive tumor margin was selected at objective magnification ×4 as previously described (9). Then, a single field of representative region presented with tumor cell in all borders of image were further chosen to assess TSP at objective magnification ×40. Whereas, biopsy sections that contained necrosis or mucin in representative region were excluded for the scoring. Subsequently, a machine algorithm based on MATLAB were used to calculate percentage of stroma of the visible field. Our previous study confirmed that assessment of TSP based on machine algorithm was more accurate than that based on artificial visualization (21). In this study, a TSP ≤50% of tumor area was categorized as low TSP, while a TSP >50% of tumor area was regarded as high TSP.

### Definitions of LODDS

LODDS was defined as the loge [(positive nodes + 0.5)/(negative nodes + 0.5)], namely, the log of the ratio between the number of positive lymph nodes and the number of negative lymph nodes (22). X-tile (Yale University, 3.6.1) was also performed to calculate the cutoff value for LODDS group. In terms of the discovery cohort and the validation cohort, LODDS was classified into three categories including ≤ −0.9138, −0.9138 to −0.2373 and > −0.2373.

### Statistical Analysis

Demographics and clinical characteristics were summarized using the average and standard deviation for continuous variables while deploying frequency and percentages for categorical variables. Continuous variables with normal distribution were compared using the Student’s t test, or the Mann-Whitney U test was used for variables with abnormal distribution. OS curves were generated by using Kaplan-Meier survival analysis, and the differences in survival distributions were performed using the log-rank test. The Cox proportional hazards model was used to determine the hazard ratio (HR) of possible risk factors and OS. Variables were converted to classify variables for univariable analysis, and the factors that showed significant associations with survival in the univariate analyses were subsequently included in the multivariate Cox regression model to identify independent prognostic factors through backward selection. All statistical analyses were performed by SPSS version 24 (IBM, Armonk, NY, USA) and R version 2.13.2 (https://www.r-project.org). Significance was set as P <0.05 in a two-sided test.

## Results

### Demographics and Clinical Characteristics of CC Patients

Based on inclusion and exclusion criteria, a total of 577 patients with colon cancer were retrospectively collected from the institutional database, including 261 patients with LSCC and 316 patients with RSCC. The demographics and clinicopathological characteristics of the entire training and validation cohorts of LSCC and RSCC were listed in **Table 1**, respectively.

**Table 1:** Demographics and clinical characteristics of CC patients.

Characteristic	LSCC				RSCC			
	Whole population	Training cohort	Validation cohort	P value	Whole population	Training cohort	Validation cohort	P value
**Age**	65.7 ± 11.1	65.90 ± 11.09	64.67 ± 11.04	0.476	67.2 ± 12.5	67.42 ± 12.45	66.29 ± 12.75	0.522
**Grade**				0.886				0.474
I	22 (8.4%)	17 (8.1%)	5 (9.6%)		26 (8.2%)	19 (7.5%)	7 (11.1%)	
II	205 (78.5%)	165 (78.9%)	40 (76.9%)		227 (71.8%)	181 (71.5%)	46 (73%)	
III	34 (13%)	27 (12.9%)	7 (13.5%)		63 (19.9%)	53 (20.9%)	10 (15.9%)	
**pTNM**				0.777				0.472
I	37 (14.2%)	30 (14.4%)	7 (13.5%)		23 (7.3%)	20 (7.9%)	3 (4.8%)	
II	121 (46.4%)	101 (48.3%)	20 (38.5%)		172 (54.4%)	142 (56.1%)	30 (47.6%)	
III	100 (38.3%)	76 (36.3%)	24 (46.2%)		118 (37.3%)	88 (34.8%)	30 (47.6%)	
IV	3 (1.1%)	2 (1%)	1 (1.9%)		3 (0.9%)	3 (1.2%)	0 (0)	
**Positive lymph node**	1.50 ± 2.94	1.38 ± 2.76	1.98 ± 3.57	0.187	2.15 ± 5.59	2.19 ± 6.03	1.97 ± 3.35	0.779
**LODDS**				0.218				0.087
≤−0.9138	178 (68.2%)	147 (70.3%)	31 (59.6%)		240 (75.9%)	196 (77.5%)	44 (69.8%)	
≤−0.2373	61 (23.4%)	44 (21.1%)	17 (32.7%)		47 (14.9%)	32 (12.6%)	15 (23.8%)	
>−0.2373	22 (8.4%)	18 (8.6%)	4 (7.7%)		29 (9.2%)	25 (9.9%)	4 (6.3%)	
**Tumor size (cm)**	3.99 ± 6.66	3.89 ± 7.40	4.36 ± 1.59	0.655	5.23 ± 2.14	5.32 ± 2.20	4.89 ± 1.89	0.16
**Ki-67**				0.268				0.047
(+, ≤25)	5 (1.9%)	2 (1%)	3 (5.8%)		10 (3.2%)	6 (2.4%)	4 (6.3%)	
(++, ≤50)	54 (20.7%)	44 (21.1%)	10 (19.2%)		66 (20.9%)	53 (20.9%)	13 (20.6%)	
(+++, ≤75)	92 (35.2%)	72 (34.4%)	20 (38.5%)		108 (34.2%)	80 (31.6%)	28 (44.4%)	
(++++, ≤100)	87 (33.3%)	72 (34.4%)	15 (28.8%)		101 (32%)	89 (35.2%)	12 (19%)	
NA	23 (8.8%)	19 (9.1%)	4 (7.7%)		31 (9.8%)	25 (9.9%)	6 (9.5%)	
**CA724**				0.433				0.269
Negative	164 (62.8%)	129 (61.7%)	35 (67.3%)		192 (60.8%)	151 (59.7%)	41 (65.1%)	
Positive	29 (11.1%)	22 (10.5%)	7 (13.5%)		51 (16.1%)	45 (17.8%)	6 (9.5%)	
N/A	68 (26.1%)	58 (27.8%)	10 (19.2%)		73 (23.1%)	57 (22.5%)	16 (25.4%)	
**CEA**				0.612				0.947
Negative	124 (47.5%)	96 (45.9%)	28 (53.8%)		160 (50.6%)	129 (51%)	31 (49.2%)	
Positive	83 (31.8%)	69 (33%)	14 (26.9%)		94 (29.7%)	75 (29.6%)	19 (30.2%)	
N/A	54 (20.7%)	44 (21.1%)	10 (19.2%)		62 (19.6%)	49 (19.4%)	13 (20.6%)	
**CA199**				0.801				0.893
Negative	186 (71.3%)	148 (70.8%)	38 (73.1%)		207 (65.5%)	167 (66%)	40 (63.5%)	
Positive	17 (6.5%)	13 (6.2%)	4 (7.7%)		45 (14.2%)	36 (14.2%)	9 (14.3%)	
N/A	58 (22.2%)	48 (23%)	10 (19.2%)		64 (20.3%)	50 (19.8%)	14 (22.2%)	
**CA50**				0.177				0.807
Negative	77 (29.5%)	56 (26.8%)	21 (40.4%)		99 (31.3%)	77 (30.4%)	22 (34.9%)	
Positive	15 (5.7%)	13 (6.2%)	2 (3.8%)		29 (9.2%)	24 (9.5%)	5 (7.9%)	
N/A	169 (64.8%)	140 (67%)	29 (55.8%)		188 (59.5%)	152 (60.1%)	36 (57.1%)	
**CA125**				0.138				0.324
Negative	94 (36%)	71 (34%)	23 (44.2%)		115 (36.4%)	89 (35.2%)	26 (41.3%)	
Positive	9 (3.4%)	6 (2.9%)	3 (5.8%)		23 (7.3%)	21 (8.3%)	2 (3.2%)	
N/A	158 (60.5%)	132 (63.2%)	26 (50%)		178 (56.3%)	143 (56.5%)	35 (55.6%)	
**TSP**				0.49				0.879
≤50%	188 (72%)	148 (70.8%)	40 (76.9%)		222 (70.3%)	177 (70%)	45 (71.4%)	
>50%	73 (28%)	61 (29.2%)	12 (23.1%)		94 (29.7%)	76 (30%)	18 (28.6%)	
**CDX2**				0.807				0.547
Negative	7 (2.7%)	5 (2.4%)	2 (3.8%)		20 (6.3%)	18 (7.1%)	2 (3.2%)	
Positive	222 (85.1%)	178 (85.2%)	44 (84.6%)		265 (83.9%)	211 (83.4%)	54 (85.7%)	
NA	32 (12.3%)	26 (12.4%)	6 (11.5%)		31 (9.8%)	24 (9.5%)	7 (11.1%)	
**MLH1**				0.536				0.618
Negative	17 (6.5%)	14 (6.7%)	3 (5.8%)		44 (13.9%)	37 (14.6%)	7 (11.1%)	
Positive	219 (83.9%)	177 (84.7%)	42 (80.8%)		244 (77.2%)	192 (75.9%)	52 (82.5%)	
NA	25 (9.6%)	18 (8.6%)	7 (13.5%)		28 (8.9%)	24 (9.5%)	4 (6.3%)	
**MSH2**				0.141				0.778
Negative	12 (4.6%)	12 (5.7%)	0 (0%)		22 (7%)	19 (7.5%)	3 (4.8%)	
Positive	223 (85.4%)	178 (85.2%)	45 (86.5%)		264 (83.5%)	209 (82.6%)	55 (87.3%)	
NA	26 (10%)	19 (9.1%)	7 (13.5%)		30 (9.5%)	25 (9.9%)	5 (7.9%)	
**ERCC1**				0.42				0.445
Negative	25 (9.6%)	22 (10.5%)	3 (5.8%)		33 (10.4%)	25 (9.9%)	8 (12.7%)	
Positive	175 (67%)	141 (67.5%)	34 (65.4%)		221 (69.9%)	175 (69.2%)	46 (73%)	
NA	61 (23.4%)	46 (22%)	15 (28.8%)		62 (19.6%)	53 (20.9%)	9 (14.3%)	
**NM23**				0.08				0.317
Negative	12 (4.6%)	7 (3.3%)	5 (9.6%)		15 (4.7%)	12 (4.7%)	3 (4.8%)	
Positive	165 (63.2%)	137 (65.6%)	28 (53.8%)		218 (69%)	170 (67.2%)	48 (76.2%)	
NA	84 (32.2%)	65 (31.1%)	19 (36.5%)		83 (26.3%)	71 (28.1%)	12 (19%)	
**COX2**				0.948				0.114
Negative	27 (10.3%)	21 (10%)	6 (11.5%)		20 (6.3%)	19 (7.5%)	1 (1.6%)	
Positive	132 (50.6%)	106 (50.7%)	26 (50%)		182 (57.6%)	140 (55.3%)	42 (66.7%)	
NA	102 (39.1%)	82 (39.2%)	20 (38.5%)		114 (36.1%)	94 (37.2%)	20 (31.7%)	
**c-MET**				0.639				0.249
Negative	62 (23.8%)	48 (23%)	14 (26.9%)		82 (25.9%)	61 (24.1%)	21 (33.3%)	
Positive	110 (42.1%)	91 (43.5%)	19 (36.5%)		138 (43.7%)	111 (43.9%)	27 (42.9%)	
NA	89 (34.1%)	70 (33.5%)	19 (36.5%)		96 (30.4%)	81 (32%)	15 (23.8%)	

In the entire group, 56.67% of patients were male, and 87% of patients were ≥60 years at diagnosis. Most patients had an adenocarcinoma histological type and moderately differentiated tumors. It was T3, T4a, and T4b tumors that accounted for 19.06, 67.59, and 0.02% of all cases, respectively. There was no significant difference between the training and validation cohorts in demographic and clinical characteristics.

### Univariate and Multivariate Analyses of Risk Factors Associated With Overall Survival

In the training cohort in LSCC, univariate analyses by Kaplan-Meier curves and log-rank tests showed that age, TNM stage, N stage, positive nodes, LODDS, and CA125 were associated with overall survival. Meanwhile, univariate analysis also showed that age, TNM stage, T stage, N stage, positive nodes, LODDS, CA724, CEA, CA199, and TSP were associated with overall survival in the training cohort of RSCC **(** **Table 2** **)**.

**Table 2:** Univariate analyses of risk factors associated with overall survival.

Variables	LSCC			RSCC		
	OS%	95% CI	P value	OS%	95% CI	P value
**Age**			0.008			0
≤67	96.10	92.35–100		87.93	81.77–94.56	
>67	85.33	78.26–93.05		73.5	66.16–81.65	
**Grade**			0.309			0.098
I	100	100–100		82.6	68.22–100	
II	90.74	86.23–95.49		83.35	77.75–89.35	
III	89.23	78.13–100		67.79	55.48–82.84	
**pTNM**			0			0.002
I	92.43	82.47–100		100	100–100	
II	99.17	97.57–100		84.72	78.48–91.44	
III	80.98()	72.44–90.53		70.71	61.82–80.88	
IV	/	/		/		
**Positive lymph node**			0			0
0	97.61	94.87–100		88	82.7–93.64	
>0	81.45	73.07–90.79		68.05	59.04–78.43	
**LODDS**			0			0
≤−0.9138	96.84	93.71–100		88.85	84.29–93.65	
≤−0.2373	87.3	77.96–97.75		64.62	49.52–84.33	
>−0.2373	57.93	38.98–86.1		30.13	15.15–59.93	
**Tumor size**			0.422			0.738
<5 cm	93.63	89.54–97–91		79.92	73.2–87.26	
≥5 cm	86.79	78.86–95.51		80.47	73.09–88.59	
**Ki-67**			0.896			0.603
(+, ≤25)	100	100–100		75	49.61–100	
(++, ≤50)	86.4	76.52–97.54		73.35	62.32–86.35	
(+++, ≤75)	94.17	89.31–99.3		79.76	70.7–89.98	
(++++, ≤100)	89.85	82.08–98.36		81.86	73.17–91.59	
NA						
**CA724**			0.386			0.039
Negative	94.69	90.82–98.73		83.44	77.23–90.14	
Positive	85.58	73.39–99.79		83.35	72.45–95.89	
**CEA**			0.48			0
Negative	94.66	90.13–99.43		89.77	84.1–95.81	
Positive	88.43	80.82–96.75		68.44	57.98–80.8	
**CA199**			0.357			0.001
Negative	93.75	89.99–97.67		85.43	79.67–91.6	
Positive	74.87	53.13–100		62.79	47.74–82.57	
**CA50**			0.759			0.275
Negative	93.47	88.10–99.17		83.61	74.91–93.31	
Positive	84	65.62–100		77.89	63.5–95.54	
**CA125**			0.002			0.164
Negative	93.91	88.72–99.41		84.63	76.73–93.34	
Positive	58.33	30–100		75.1	57.63–97.86	
**TSP**			0.508			0.004
≤50%	92.11	87.72–96.71		84.33	78.69–90.38	
>50%	90.05	82.71–98.03		70.56	60.85–81.83	
**CDX2**			0.109			0.585
Negative	85.71	63.34–100		79.06	62.7–99.69	
Positive	93.41	89.72–97.25		79.57	73.9–85.68	
**MLH1**			0.98			0.678
Negative	86.88	71.35–100		85.26	74.99–96.92	
Positive	92.03	87.94–96.31		79.51	73.44–86.08	
**MSH2**			0.744			0.998
Negative	100	100–100		89.64	76.95–100	
Positive	89.96	85.31–94.85		79.23	73.45–85.46	
**ERCC1**			0.502			0.7
Negative	90	77.77–100		77.59	63.15–95.31	
Positive	90.24	85.32–95.44		82.24	76.66–88.24	
**NM23**			0.91			0.068
Negative	83.33	64.7–100		71.79	51.77–99.57	
Positive	91.84	87.15–96.8		80.43	74.51–86.83	
**COX2**			0.408			0.613
Negative	96	88.62–100		78.75	62.2–99.7	
Positive	90.4	84.25–97		83.53	77.47–90.06	
**c-MET**			0.78			0.694
Negative	91.85	85.26–98.96		82.58	74.69–91.31	
Positive	88.74	81.28–96.88		76.32	67.55–86.22	

Multivariate analysis showed that only age, LODDS, and CA125 were independent risk factors for overall survival in patients with LSCC, and age, LODDS, and TSP were independent risk factors for overall survival in patients with RSCC (**Tables 3**, **4**).

**Table 3:** Multivariate Cox regression analyses of risk factors associated with overall survival in LSCC.

Variables	HR	95% CI	P value
**CA125 >35 U/ml**	5.561	1.533–20.17	0.009
**Age >67**	3.317	1.257–8.751	0.015
**LODDS**			
(−0.9138)–(−0.2373)	4.484	1.691–14.693	0.004
>−0.2373	11.868	3.855–36.537	0

**Table 4:** Multivariate Cox regression analyses of risk factors associated with overall survival in RSCC.

Variable	HR	95%	P value
**TSP**	2.919	1.59–5.358	0.001
**Age >67**	4.906	2.319–10.378	0
**LODDS**			
(−0.9138)–(−0.2373)	2.475	1.098–5.581	0.029
>−0.2373	11.204	5.288–23.735	0

### Construction and Validation of the Nomogram

**Figure 2:**
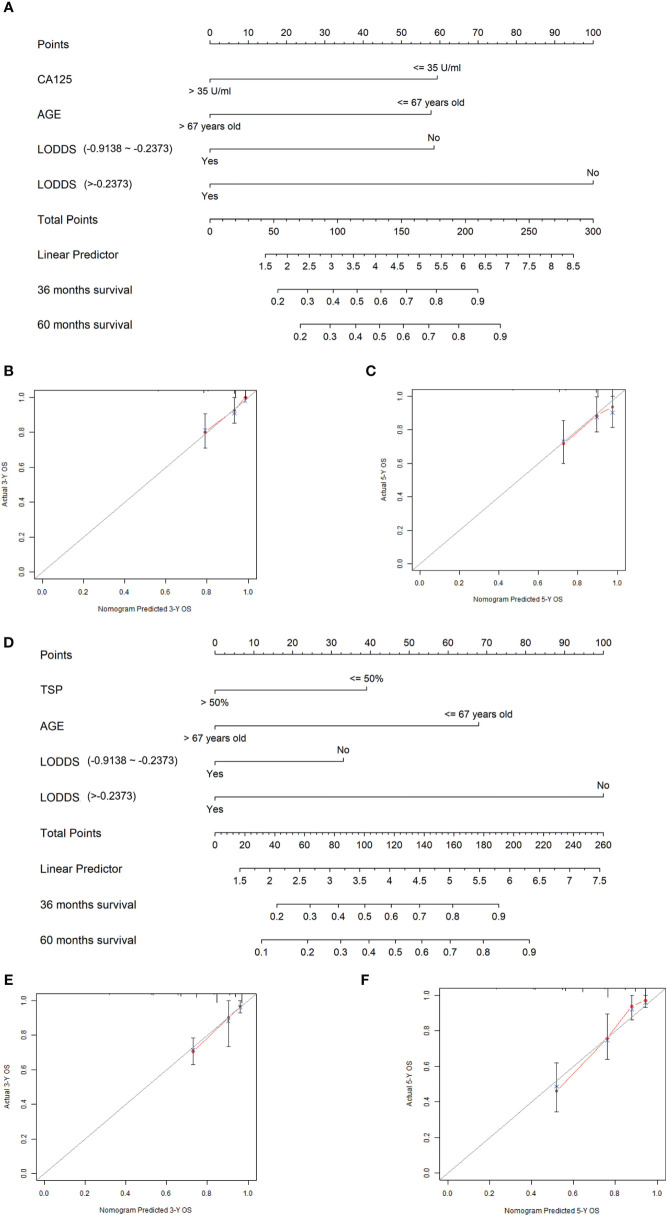
Construction and validation of the nomogram. **(A)** The nomogram to predict 3-year and 5-year survival probability for patients with LSCC. **(B, C)** The calibration curves of 3- and 5-year survival probability in LSCC patients. **(D)** The nomogram to predict 3-year and 5-year survival probability for patients with RSCC. **(E, F)** The calibration curves of 3- and 5-year survival probability in RSCC patients.

Based on the multivariate Cox regression analysis results, age, LODDS, and CA125 were defined as independent prognostic factors in LSCC, and these were integrated to develop the nomogram of LSCC (**Figures 2A**–**C**). Similarly, age, LODDS, and TSP were integrated to construct nomogram of RSCC (**Figures 2D**–**F**).

According to the nomogram of LSCC, LODDS had the greatest influence on the prognosis of LSCC, followed by CA125. While in the nomogram of RSCC, LODDS and TSP played crucial roles in the prognosis of RSCC. The total score based on individual scores of those eight parameters and a particular probability of 3- and 5-year OS could be worked out by clinicians.

To confirm that the corresponding nomograms prediction model had higher efficacy in predicting the prognosis of LSCC and RSCC patients than TNM classification, we compared C-index among training cohort, validation cohort, and whole cohort in LSCC and RSCC, respectively. In LSCC nomogram, the C-indexes in the training and validation groups were 0.837, 0.942, and 0.837 and 0.790, 0.821, and 0.780, respectively, compared with C-indexes of 0.756, 0.768, and 0.747 and 0.631, 0.624, and 0.629, respectively, based on TNM classification (**Tables 5** and **6**), which showed that the simple-to-use nomogram was expected to be more accurate than TNM stage. In addition, calibration curves for the nomogram showed no deviations from the reference line, which meant a high degree of reliability (23) (**Figure 2**).

**Table 5:** Comparison between nomogram and TNM classification in LSCC.

Variables	C-index	95% CI	P value
**Derivation cohort**			0.005
Nomogram	0.837	0.826–0.848	
TNM stage	0.756	0.744–0.767	
**Validation cohort**			<0.001
Nomogram	0.942	0.922–0.962	
TNM stage	0.768	0.746–0.790	
**Whole cohort**			<0.001
Nomogram	0.837	0.827–0.846	
TNM stage	0.747	0.737–0.756	

**Table 6:** Comparison between nomogram and TNM classification in RSCC.

Variables	C-index	95% CI	P value
**Derivation cohort**			<0.001
Nomogram	0.79	0.781–0.799	
TNM stage	0.631	0.622–0.640	
**Validation cohort**			<0.001
Nomogram	0.821	0.798–0.844	
TNM stage	0.624	0.590–0.658	
**Whole cohort**			<0.001
Nomogram	0.78	0.773–0.787	
TNM stage	0.629	0.622–0.636	

### Risk Stratification Based on the Nomogram

**Figure 3:**
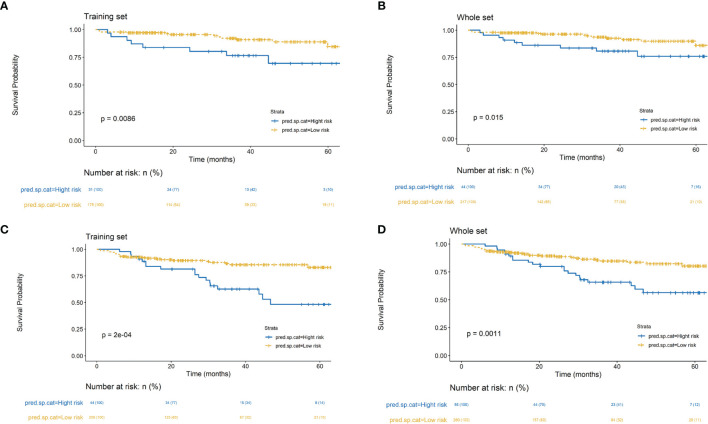
Kaplan-Meier survival curves of risk groups stratified based on the nomogram for LSCC training set **(A)** and whole set **(B)**. Kaplan-Meier survival curves of risk groups stratified based on the nomogram for RSCC training set **(C)** and whole set **(D)**.

The cutoff values were given out by dividing all patients in the training and whole cohorts into two subgroups based on the total score, in which each group represented a distinct prognosis in LSCC nomogram and RSCC nomogram, respectively. The Kaplan-Meier survival curves were subsequently delineated and shown in **Figure 3**. In the training and whole cohorts of LSCC, Kaplan-Meier survival curve analysis showed the two groups had statistically different prognosis in both train cohort and whole cohorts. As for the nomogram of RSCC, in the training and whole cohorts of RSCC, Kaplan-Meier survival curve analysis also showed the two groups had statistically different prognosis in both train cohort and whole cohorts.

### Clinical Value of the Nomogram Compared With 8th AJCC TMN

**Figure 4:**
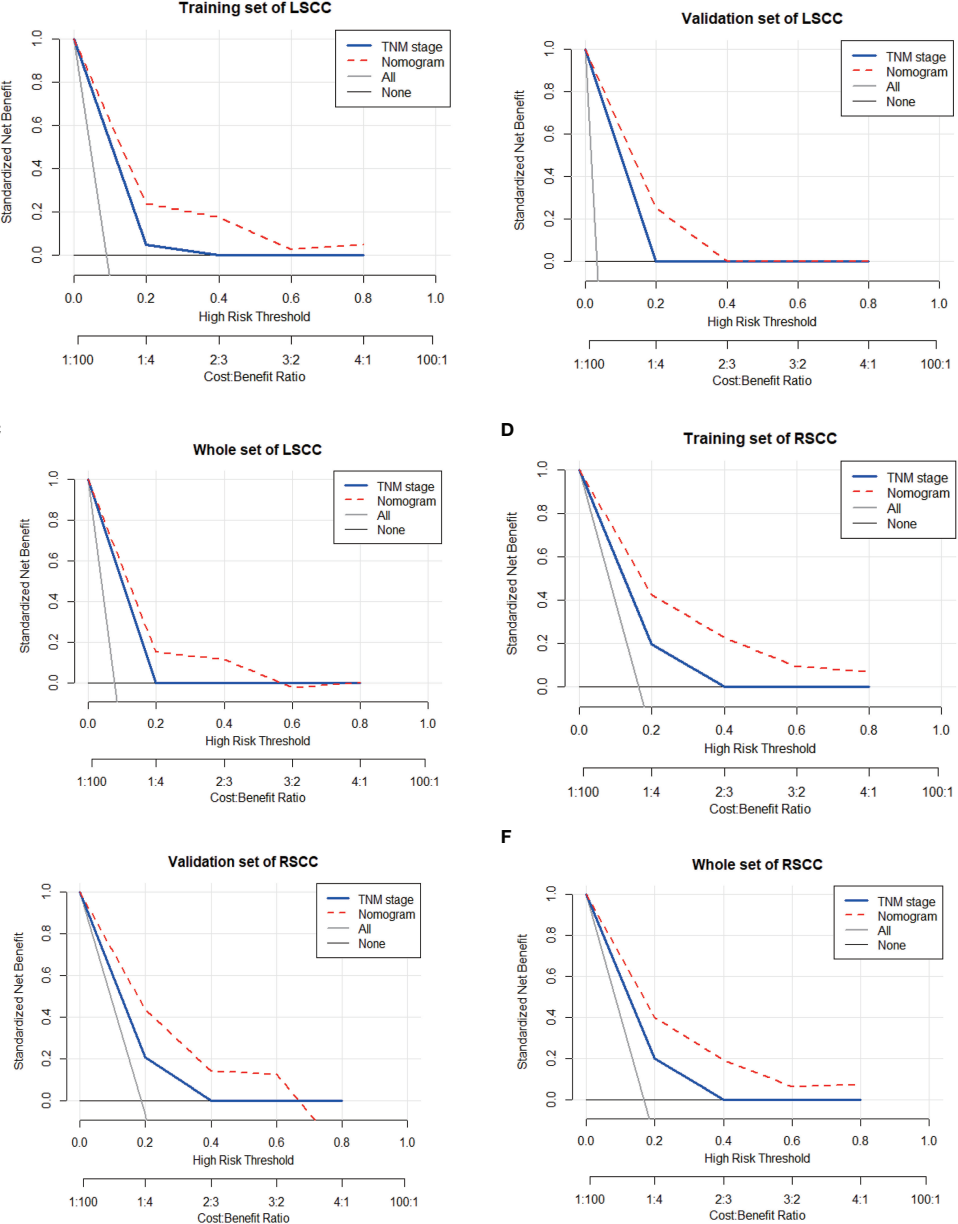
Decision curve analysis (DCA) to assess the clinical usefulness of the nomogram and TNM stage in the training set **(A)**, validation set **(B)**, and whole set of LSCC **(C)**. And DCA in the training set **(D)**, validation set **(E)**, and whole set of RSCC **(F)**.

DCA is a novel method for evaluating alternative prognostic strategies, which has advantages over area under curve (AUC) (24). DCA curves for the novel nomogram of LSCC and RSCC and TNM classification in the training, validation, and the entire groups are presented in **Figure 4**, respectively. Compared with TNM classification, DCA of the nomogram had higher net benefits, which indicated that the nomogram had better clinical utility than TNM classification.

### Development of Webserver for Easy Access of Nomogram

**Figure 5:**
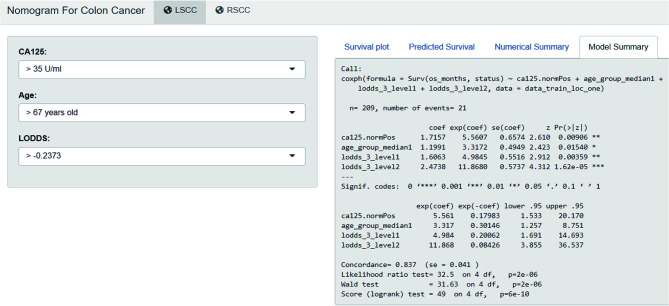
Development of webserver for easy access of nomogram (https://colon-cancer-prediction-tool.shinyapps.io/nomogram_for_colon_cancer/).

An online version of our nomogram (**Figure 5**) can be accessed at https://colon-cancer-prediction-tool.shinyapps.io/nomogram_for_colon_cancer/, to assist researchers and clinicians. Predicted survival probability across time can be easily determined by inputting clinical features and reading output figures and tables generated by the webserver.

## Discussion

In the present study, we developed and validated personalized nomograms incorporating age, CA125, LODDS, and TSP to predict the OS probability for LSCC patients and RSCC patients after radical resection, respectively. The nomograms had exhibited more competitive capability of discrimination and calibration in both of the training and validation cohorts. From the point of clinical application, DCA analysis revealed it had promising clinical applicability, and C-index analysis demonstrated nomograms had superior prognostication performance compared to the 8^th^ AJCC TNM classification (0.837 *vs.* 0.747, and 0.780 *vs.* 0.629). Thus, the constructed nomograms were able to provide a feasible and customized tool to inform patients about their long-term prognoses and help clinicians to make more individual treatment decisions.

Radical resection was considered to be the only curative approach for CC patients (25). Many patients could obtain 5-year survival rate range from 60 to 79%, revealing the prognostic heterogeneity associated with this disease (26). In regard to prognostic heterogeneity, recent study demonstrated that colon cancer side should be acknowledged as a criterion for prognosis (14). Thus, we established different nomograms based on colon cancer side to deliver more customized prognosis prediction. Besides, it was found that the more the proportion of tumor stroma had increased, so much the poorer survival in patients with solid tumor including colon cancer (27, 28). In spite of nomograms for survival prediction of colon cancer patients were proposed previously (29, 30), the nomogram adopting TSP as an independent prognostic factor is firstly brought forward by our group as far as we are concerned. Interestingly, our results showed TSP was higher in RSCC compared to LSCC, and univariate and multivariate analyses further demonstrated TSP acted as an independent prognostic factor in RSCC. Indeed, given that the present threshold of 50% TSP was consistent with previous studies, these results suggested that this simple, rapid assessment of the tumor stroma using machine algorithm might improve prognostic prediction in CC patients (9, 10, 31). Despite recognition of the importance of the TSP in prognostic prediction, its differences in LSCC and RSCC have yet to be fully investigated. First, from a clinical standpoint, RSCC patients are more likely to exhibit advanced tumor stage and show poor prognosis and overall survival, which is consistent with poor prognosis of higher TSP (14). Second, from a biological viewpoint, the capacity of RSCC to detoxify carcinogens is weaker than that of LSCC, owing to the fact that stroma, a target of carcinogens, modulates the growth and oncogenic potential of adjacent epithelium (32, 33).

To date, there are several established nomograms that are capable of predicting the survival after radical resection for CC patients (20, 29, 34–38). Some researches reported that the prognosis of CC was obviously connected with factors involving age, sex, and tumor size, while others showed the prognosis of CC was irrelevant to the above factors (20, 34, 35). Mo et al. recently constructed and validated prognostic nomograms incorporating demographic information, clinicopathological features, and distant metastasis status to predict OS in CRC patients, with AUCs of 0.764, 0.762, and 0.745 in 1-, 3-, 5-year OS, respectively (20). Sjoquist et al. conducted the ARCAD nomogram project to develop a nomogram to predict OS in advanced CRC patients who received first-line systemic therapy. Final nomograms were well calibrated and internally and externally valid, with a high predictive performance (C=0.68) (29). Besides traditional indicators, recent studies incorporated novel prognostic factors including tumor deposits, CpG sites, and autophagy signature genes into nomograms, respectively, which also showed the potential to assess CRC patient prognosis (36–38). Simultaneously, almost all of them were focused on CRC patients of all sides (LSCC, RSCC, and rectal cancer). However, multiple studies reported biological and survival differences between right- and left-sided colon cancer, which might release a signal corresponding nomogram for RSCC and LSCC patients was called for separate and further research (12–16, 39).

Tumors arising on the right side of the colon, in fact, were seemed to follow different molecular pathways of oncogenesis with LSCC (40). RSCCs were more commonly diploid and characterized by mucinous histology, high microsatellite instability, CpG island methylation, and BRAF mutation. Conversely, LSCCs were found to have frequent p53 and KRAS mutations (13, 40). Apart from intrinsic biological differences (i.e., higher rate of BRAF mutant cases) related to a more aggressive clinical behavior, several other factors including surgical technique and sensitivity to chemotherapy should be taken into account to explain the different outcomes in LSCC and RSCC (14, 40). Petrelli F et al. demonstrated that tumor location had a critical role in determining CC prognosis, being a surrogate of different and poor biology (14). Shida D et al. conducted a nationwide multicenter retrospective study and found RSCC patients had worse OS comparing to LSCC patients (39). Consistent with previous studies, our research also showed LSCC patients had better survival benefits than RSCC patients. As a result, we developed and validated corresponding nomograms for LSCC and RSCC patients, to achieve better personalized prediction.

Although some nomograms have been developed to predict individual survival probabilities for patients with CRC, most nomograms were focused on general factors including age, sex, usual hematological indexes, and common clinicopathological characteristics (20, 34, 35). Whereas, there are some unique points including TSP and LODDS in our nomograms. To achieve the best of comprehensiveness and comprehensibility in the nomograms is inevitable and elusive goal for researchers. However, the applicable target of our nomogram is relatively comprehensive and individualized, involving LSCC and RSCC, respectively. Additionally, improved accuracy of nomograms sometimes comes at the cost of increased complexity of the nomogram (41). Our nomograms are concise, with only three predictive factors both in LSCC and RSCC nomograms, yet remain accurate. All the clinical parameters needed for our nomograms are available after surgical resection and routine pathologic examination, without adding any further burden to patients.

Though our nomograms demonstrated satisfactory performance in predicting individual survival probability for patients with CC after surgery resection, our study did have several limitations. First, our data were of limited size and derived from data collected at a single institution, and the follow-up missing patients were relatively large, which limited the generalizability and applicable scope of the nomograms. Secondly, our nomograms were mainly based on pathological outcomes; therefore, it is inapplicable to evaluate non-surgical patients. Third, although the model still worked well in our internal cohort, which was intended for relatively strict validation, multi-institutional, prospective validation would provide more convincing evidence.

## Conclusion

In summary, we have established and validated original predictive nomograms for the survival of patients with LSCC and RSCC after surgery respectively, providing individualized outcome predictions with good accuracy, reliability, availability, and applicability. These convenient nomograms could be helpful to clinicians and patients in the treatment decision-making process.

## Data Availability Statement

The raw data supporting the conclusions of this article will be made available by the authors, without undue reservation.

## Ethics Statement

The studies involving human participants were reviewed and approved by the Clinical Research Ethics Committee of Shanghai General Hospital. Written informed consent for participation was not required for this study in accordance with the national legislation and the institutional requirements.

## Author Contributions

Study design: CH, ZQ, QL, and ZL. Data collection: ZL, ZF TL, and YZ. Manuscript preparation: CH and ZL. Data analysis: ZL, JZ, YY, ZY, QL, and ZQ. All authors are also responsible for the manuscript content. All authors contributed to the article and approved the submitted version.

## Funding

This work was supported by the National Natural Science Foundation of China (Grant No. 82072662 and 81772526), Shanghai Municipal Education Commission-Gaofeng Clinical Medicine Grant Support (20191425), Shanghai Jiaotong University Medical Cross Fund (YG2017MS28), Shanghai Municipal Science and Technology Committee (14411966800), Science and Technology Commission Project of Songjiang District (18SJKJGG23 and 19SJKJGG22), Three-year Action Plan for Clinical Skills and Clinical Innovation in Shanghai-level Hospitals (SHDC2020CR4022), and 2021 Shanghai "Rising Stars of Medical Talent" Youth Development Program: Outstanding Youth Medical Talents. No funders have any roles in study design, data collection and analysis, decision to publish, or preparation of the manuscript.

## Conflict of Interest

The authors declare that the research was conducted in the absence of any commercial or financial relationships that could be construed as a potential conflict of interest.

## Publisher’s Note

All claims expressed in this article are solely those of the authors and do not necessarily represent those of their affiliated organizations, or those of the publisher, the editors and the reviewers. Any product that may be evaluated in this article, or claim that may be made by its manufacturer, is not guaranteed or endorsed by the publisher.

## References

[1] Bray F Ferlay J Soerjomataram I Siegel RL Torre LA Jemal A. Global Cancer Statistics 2018: GLOBOCAN Estimates of Incidence and Mortality Worldwide for 36 Cancers in 185 Countries. CA Cancer J Clin (2018) 68(6):394–424. doi: 10.3322/caac.21492 30207593

[2] Jung G Hernández-Illán E Moreira L Balaguer F Goel A. Epigenetics of Colorectal Cancer: Biomarker and Therapeutic Potential. Nat Rev Gastroenterol Hepatol (2020) 17(2):111–30. doi: 10.1038/s41575-019-0230-y PMC7228650 31900466

[3] Dienstmann R Mason MJ Sinicrope FA Phipps AI Tejpar S Nesbakken A. Prediction of Overall Survival in Stage II and III Colon Cancer Beyond TNM System: A Retrospective, Pooled Biomarker Study. Ann Oncol (2017) 28(5):1023–31. doi: 10.1093/annonc/mdx052 PMC5406760 28453697

[4] Piñeros M Parkin DM Ward K Chokunonga E Ervik M Farrugia H. Essential TNM: A Registry Tool to Reduce Gaps in Cancer Staging Information. Lancet Oncol (2019) 20(2):e103–11. doi: 10.1016/S1470-2045(18)30897-0 30712797

[5] Chu QD Zhou M Medeiros K Peddi P. Positive Surgical Margins Contribute to the Survival Paradox Between Patients With Stage IIB/C (T4N0) and Stage IIIA (T1-2n1, T1N2a) Colon Cancer. Surgery (2016) 160(5):1333–43. doi: 10.1016/j.surg.2016.05.028 27425043

[6] Kim MJ Jeong SY Choi SJ Ryoo SB Park JW Park KJ. Survival Paradox Between Stage IIB/C (T4N0) and Stage IIIA (T1-2N1) Colon Cancer. Ann Surg Oncol (2015) 22(2):505–12. doi: 10.1245/s10434-014-3982-1 25145501

[7] Kattan MW Hess KR Amin MB Lu Y Moons KG Gershenwald JE. American Joint Committee on Cancer Acceptance Criteria for Inclusion of Risk Models for Individualized Prognosis in the Practice of Precision Medicine. CA Cancer J Clin (2016) 66(5):370–4. doi: 10.3322/caac.21339 PMC4955656 26784705

[8] Weiser MR Gönen M Chou JF Kattan MW Schrag D. Predicting Survival After Curative Colectomy for Cancer: Individualizing Colon Cancer Staging. J Clin Oncol (2011) 29(36):4796–802. doi: 10.1200/JCO.2011.36.5080 PMC3664036 22084366

[9] Park JH Richards CH McMillan DC Horgan PG Roxburgh CSD. The Relationship Between Tumour Stroma Percentage, the Tumour Microenvironment and Survival in Patients With Primary Operable Colorectal Cancer. Ann Oncol (2014) 25(3):644–51. doi: 10.1093/annonc/mdt593 PMC4433525 24458470

[10] Zhang QW Zhang CH Pan YB Biondi A Fico V Persiani R. Prognosis of Colorectal Cancer Patients is Associated With the Novel Log Odds of Positive Lymph Nodes Scheme: Derivation and External Validation. J Cancer (2020) 11(7):1702–11. doi: 10.7150/jca.38180 PMC7052858 32194782

[11] Huijbers A Tollenaar RA v Pelt GW Zeestraten EC Dutton S McConkey CC. The Proportion of Tumor-Stroma as a Strong Prognosticator for Stage II and III Colon Cancer Patients: Validation in the VICTOR Trial. Ann Oncol (2013) 24(1):179–85. doi: 10.1093/annonc/mds246 22865778

[12] Baran B Mert Ozupek N Yerli Tetik N Acar E Bekcioglu O Baskin Y. Difference Between Left-Sided and Right-Sided Colorectal Cancer: A Focused Review of Literature. Gastroenterol Res (2018) 11(4):264–73. doi: 10.14740/gr1062w PMC6089587 30116425

[13] Imperial R Ahmed Z Toor OM Erdoğan C Khaliq A Case P. Comparative Proteogenomic Analysis of Right-Sided Colon Cancer, Left-Sided Colon Cancer and Rectal Cancer Reveals Distinct Mutational Profiles. Mol Cancer (2018) 17(1):177. doi: 10.1186/s12943-018-0923-9 30577807 PMC6303985

[14] Petrelli F Tomasello G Borgonovo K Ghidini M Turati L Dallera P. Prognostic Survival Associated With Left-Sided *vs* Right-Sided Colon Cancer: A Systematic Review and Meta-Analysis. JAMA Oncol (2017) 3(2):211–9. doi: 10.1001/jamaoncol.2016.4227 27787550

[15] Lee MS Menter DG Kopetz S. Right *Versus* Left Colon Cancer Biology: Integrating the Consensus Molecular Subtypes. J Natl Compr Canc Netw (2017) 15(3):411–9. doi: 10.6004/jnccn.2017.0038 28275039

[16] Gurzu S Jung J Azamfirei L Mezei T Cîmpean AM Szentirmay Z. The Angiogenesis in Colorectal Carcinomas With and Without Lymph Node Metastases. Rom J Morphol Embryol (2008) 49(2):149–52. 18516319

[17] Iasonos A Schrag D Raj GV Panageas KS. How to Build and Interpret a Nomogram for Cancer Prognosis. J Clin Oncol (2008) 26(8):1364–70. doi: 10.1200/JCO.2007.12.9791 18323559

[18] Mahar AL Compton C Halabi S Hess KR Weiser MR Groome PA. Personalizing Prognosis in Colorectal Cancer: A Systematic Review of the Quality and Nature of Clinical Prognostic Tools for Survival Outcomes. J Surg Oncol (2017) 116(8):969–82. doi: 10.1002/jso.24774 PMC5760443 28767139

[19] Pan YX Chen JC Fang AP Wang XH Chen JB Wang JC. A Nomogram Predicting the Recurrence of Hepatocellular Carcinoma in Patients After Laparoscopic Hepatectomy. Cancer Commun (Lond) (2019) 39(1):55. doi: 10.1186/s40880-019-0404-6 31601270 PMC6788088

[20] Mo S Cai X Zhou Z Li Y Hu X Ma X. Nomograms for Predicting Specific Distant Metastatic Sites and Overall Survival of Colorectal Cancer Patients: A Large Population-Based Real-World Study. Clin Transl Med (2020) 10(1):169–81. doi: 10.1002/ctm2.20 PMC7240852 32508027

[21] Li T Yu Z Yang Y Fu Z Chen Z Li Q. Rapid Multi-Dynamic Algorithm for Gray Image Analysis of the Stroma Percentage on Colorectal Cancer. J Cancer (2021) 12(15):4561–73. doi: 10.7150/jca.58887 PMC8210572 34149920

[22] Baqar AR Wilkins S Wang W Oliva K McMurrick P. Log Odds of Positive Lymph Nodes Is Prognostically Equivalent to Lymph Node Ratio in Non-Metastatic Colon Cancer. BMC Cancer (2020) 20(1):762. doi: 10.1186/s12885-020-07260-y 32795292 PMC7427861

[23] Konishi T Shimada Y Hsu M Wei IH Pappou E Smith JJ. Contemporary Validation of a Nomogram Predicting Colon Cancer Recurrence, Revealing All-Stage Improved Outcomes. JNCI Cancer Spectr (2019) 3(2):pkz015. doi: 10.1093/jncics/pkz015 31119207 PMC6512350

[24] Mo S Zhou Z Dai W Xiang W Han L Zhang L. Development and External Validation of a Predictive Scoring System Associated With Metastasis of T1-2 Colorectal Tumors to Lymph Nodes. Clin Transl Med (2020) 10(1):275–87. doi: 10.1002/ctm2.30 PMC7240869 32508061

[25] Kuipers EJ Grady WM Lieberman D Seufferlein T Sung JJ Boelens PG. Colorectal Cancer. Nat Rev Dis Primers (2015) 1:15065. doi: 10.1038/nrdp.2015.65 27189416 PMC4874655

[26] Brajcich BC Stulberg JJ Palis BE Chung JW Huang R Nelson H. Association Between Surgical Technical Skill and Long-Term Survival for Colon Cancer. JAMA Oncol (2020) 7(1):127–9. doi: 10.1001/jamaoncol.2020.5462 PMC7600051 33125472

[27] Wu J Liang C Chen M Su W. Association Between Tumor-Stroma Ratio and Prognosis in Solid Tumor Patients: A Systematic Review and Meta-Analysis. Oncotarget (2016) 7(42):68954–65. doi: 10.18632/oncotarget.12135 PMC5356603 27661111

[28] Reichling C Taieb J Derangere V Klopfenstein Q Le Malicot K Gornet JM. Artificial Intelligence-Guided Tissue Analysis Combined With Immune Infiltrate Assessment Predicts Stage III Colon Cancer Outcomes in PETACC08 Study. Gut (2020) 69(4):681–90. doi: 10.1136/gutjnl-2019-319292 PMC7063404 31780575

[29] Sjoquist KM Renfro LA Simes RJ Tebbutt NC Clarke S Seymour MT. Personalizing Survival Predictions in Advanced Colorectal Cancer: The ARCAD Nomogram Project. J Natl Cancer Inst (2018) 110(6):638–48. doi: 10.1093/jnci/djx253 PMC6005015 29267900

[30] Weiser MR Landmann RG Kattan MW Gonen M Shia J Chou J. Individualized Prediction of Colon Cancer Recurrence Using a Nomogram. J Clin Oncol (2008) 26(3):380–5. doi: 10.1200/JCO.2007.14.1291 18202413

[31] Mesker WE Junggeburt JM Szuhai K de Heer P Morreau H Tanke HJ. The Carcinoma-Stromal Ratio of Colon Carcinoma is an Independent Factor for Survival Compared to Lymph Node Status and Tumor Stage. Cell Oncol (2007) 29(5):387–98. doi: 10.1155/2007/175276 PMC4617992 17726261

[32] Fukino K Shen L Matsumoto S Morrison CD Mutter GL Eng C. Combined Total Genome Loss of Heterozygosity Scan of Breast Cancer Stroma and Epithelium Reveals Multiplicity of Stromal Targets. Cancer Res (2004) 64(20):7231–6. doi: 10.1158/0008-5472.CAN-04-2866 15492239

[33] Maffini MV Soto AM Calabro JM Ucci AA Sonnenschein C. The Stroma as a Crucial Target in Rat Mammary Gland Carcinogenesis. J Cell Sci (2004) 117(Pt 8):1495–502. doi: 10.1242/jcs.01000 14996910

[34] Hong T Cai D Jin L Zhang Y Lu T Hua D. Development and Validation of a Nomogram to Predict Survival After Curative Resection of Nonmetastatic Colorectal Cancer. Cancer Med (2020) 9(12):4126–36. doi: 10.1002/cam4.3010 PMC7300391 32314876

[35] Liu J Huang X Yang W Li C Li Z Zhang C. Nomogram for Predicting Overall Survival in Stage II-III Colorectal Cancer. Cancer Med (2020) 9(7):2363–71. doi: 10.1002/cam4.2896 PMC7131840 32027098

[36] Bai R Tan Y Li D Yang M Yu L Yuan Y. Development and Validation of a Novel Prognostic Nomogram Including Tumor Deposits Could Better Predict Survival for Colorectal Cancer: A Population-Based Study. Ann Transl Med (2021) 9(8):620. doi: 10.21037/atm-20-4728 33987318 PMC8106036

[37] Wang X Wang D Liu J Feng M Wu X. A Novel CpG-Methylation-Based Nomogram Predicts Survival in Colorectal Cancer. Epigenetics (2020) 15(11):1213–27. doi: 10.1080/15592294.2020.1762368 PMC7595584 32396412

[38] Zhao H Huang C Luo Y Yao X Hu Y Wang M. A Correlation Study of Prognostic Risk Prediction for Colorectal Cancer Based on Autophagy Signature Genes. Front Oncol (2021) 11:595099. doi: 10.3389/fonc.2021.595099 34168974 PMC8218632

[39] Shida D Inoue M Tanabe T Moritani K Tsukamoto S Yamauchi S. Prognostic Impact of Primary Tumor Location in Stage III Colorectal Cancer-Right-Sided Colon *Versus* Left-Sided Colon *Versus* Rectum: A Nationwide Multicenter Retrospective Study. J Gastroenterol (2020) 55(10):958–68. doi: 10.1007/s00535-020-01706-7 32651860

[40] Mukund K Syulyukina N Ramamoorthy S Subramaniam S. Right and Left-Sided Colon Cancers -Specificity of Molecular Mechanisms in Tumorigenesis and Progression. BMC Cancer (2020) 20(1):317. doi: 10.1186/s12885-020-06784-7 32293332 PMC7161305

[41] Dong F Shen Y Gao F Shi X Xu T Wang X. Nomograms to Predict Individual Prognosis of Patients With Primary Small Cell Carcinoma of the Bladder. J Cancer (2018) 9(7):1152–64. doi: 10.7150/jca.23344 PMC5907663 29675096

